# Mechanisms of Myocardial Injury in COVID-19

**DOI:** 10.1093/clinchem/hvab111

**Published:** 2021-07-05

**Authors:** Anda Bularga, Andrew R Chapman, Nicholas L Mills

**Affiliations:** 1 BHF Centre for Cardiovascular Science, University of Edinburgh, Edinburgh, UK; 2 Usher Institute of Population Health Sciences and Informatics University of Edinburgh, Edinburgh, UK

**Keywords:** COVID-19, high-sensitivity cardiac troponin, myocardial injury

Almost 18 months following its first report, coronavirus disease 2019 or COVID-19 continues to cause fatalities worldwide and disrupt all aspects of daily life (1). Our knowledge of this condition, the mechanisms of severe acute respiratory syndrome coronavirus-2 (SARS-CoV-2) infection and its systemic consequences have improved through an unprecedented global effort from the diagnostic, medical, and public health research community (1). As a consequence, effective treatments (2) and vaccines (3) have been developed, tested, and implemented within record timeframes to mitigate some of the critical effects of the COVID-19 illness.

From the early days of the pandemic, reports of increased cardiac biomarkers (cardiac troponin and natriuretic peptides) in patients hospitalized with COVID-19 have emerged (4). With time it became increasingly recognized that myocardial injury as measured by increased cardiac troponin concentrations, was associated with severe COVID-19 illness and worse clinical outcomes (5). While early guidance from international cardiology societies (5, 6) recommended limiting cardiac biomarker testing to patients with COVID-19 and symptoms of suspected acute coronary syndrome or heart failure, many institutions worldwide implemented routine testing as a simple, inexpensive, bedside tool for risk stratification in COVID-19 patients. Data from New York (7), Wuhan (8), and many other cities were consistent with these early reports and found a strong independent association between increased cardiac troponin and in-hospital mortality (9). Despite major efforts to evaluate the cardiac implications of COVID-19, so far very little is known about the underlying mechanism of myocardial injury and whether we can modify this to improve clinical outcomes.

In the current issue of *Clinical Chemistry*, De Michieli and colleagues report the first systematic adjudication of the underlying mechanisms of myocardial injury in COVID-19 (10). In a US cohort of 367 patients with COVID-19 who underwent high-sensitivity cardiac troponin T testing at the clinician’s discretion, 46% (169/367) of the patients had at least one measure above the sex-specific 99th percentile on initial or serial testing. The authors performed clinical adjudication according to the Fourth Universal Definition of Myocardial Infarction using all available medical record data, including observations and cardiac investigations such as the electrocardiogram, echocardiogram, and coronary imaging. Their findings are striking, but perhaps not unexpected. Nineteen out of every twenty patients with COVID-19 and myocardial injury did not have evidence of myocardial ischemia or met diagnostic criteria for type 1 or 2 myocardial infarction. Most had non-ischemic acute myocardial injury due to critical illness or chronic myocardial injury due to comorbid conditions such as chronic heart failure, cardiomyopathy, or kidney disease. Furthermore, cardiac causes of acute myocardial injury, such as myocarditis, were seldomly identified in this patient cohort. These are important findings. While multiple cardiac phenotypes as direct consequences of the SARS-CoV-2 viral infection on the myocardium and circulatory system have been described, these account for the minority of cases of myocardial injury in patients with COVID-19. The high number of increased cardiac troponin concentrations in COVID-19 patients most likely reflects the severity of the acute illness and increased likelihood of severe illness in patients with pre-existing cardiovascular disease.

In addition to their careful diagnostic adjudication, the strengths of De Michieli’s work lie in the consistent use of a high-sensitivity cardiac troponin assay and more importantly the recommended sex-specific 99th percentile to define myocardial injury. This approach has been shown to improve the detection of myocardial injury particularly in women (11). Furthermore, two-thirds of patients underwent serial testing with two or more troponin tests. The authors explored a range of relevant outcomes including short-term all-cause mortality and major adverse events as a composite of short-term mortality, respiratory, and cardiac complications. Consistent with previous literature (7–9), myocardial injury was associated with higher incidence of in-hospital mortality or death at 30 days post discharge, and patients with myocardial injury were more likely to have respiratory or cardiac complications, such as respiratory failure requiring ventilation, acute respiratory distress syndrome, and heart failure, compared to patients without myocardial injury. In an adjusted analysis myocardial injury was an independent predictor of major adverse events (adjusted OR 3.84, 95% CI 2.00–7.36), but not of short-term mortality (in-hospital death or death within 30 days posthospital discharge) (adjusted HR 1.56, 95% CI 0.68–3.57) reflecting the relatively small sample size.

The work of De Michieli and colleagues confirm previous findings that high-sensitivity cardiac troponin is a powerful prognostic marker in the context of severe COVID-19 illness and adds some novel evidence on risk stratification. As in other areas of clinical practice, such as the evaluation of patients with suspected acute coronary syndrome (12), very low high-sensitivity troponin concentrations—below the limit of quantification—provide substantial reassurance about prognosis. One in 4 patients with COVID-19 had unquantifiable cardiac troponin concentrations and, in this group, there were 0 deaths in hospital or at 30 days post discharge. This observation is entirely plausible given this group were younger with a lower prevalence of cardiovascular risk factors compared to those with quantifiable cardiac troponin values or myocardial injury. It is increasingly clear that high-sensitivity troponin is a dynamic barometer of cardiac health providing powerful risk prediction beyond the acute coronary syndrome (13). This knowledge could help improve the triage and risk stratification of patients, potentially avoiding unnecessary cardiac screening during acute illness or later in the recovery period.

Compelling data have shown strong links between increased high-sensitivity cardiac troponin concentrations and adverse clinical outcomes in conditions other than an acute coronary syndrome. However, we should highlight the paucity of evidence to guide investigation and management in patients identified with myocardial injury. It is currently unknown whether routine testing to screen for underlying heart disease has an overall benefit. Given there were few cases of myocardial infarction or viral myocarditis, would care or outcomes have differed if these patients had not undergone cardiac troponin testing? Future research should focus on determining whether these prognostic insights can translate into better care and improved outcomes for patients.

This study has some limitations, most of which have been acknowledged by the authors. As with many of the studies published in this area, this study was retrospective and observational where the selection of patients for cardiac troponin testing will have introduced an important bias. It is therefore not possible to accurately determine the true prevalence of COVID-19-associated myocardial injury. So far, there have been no prospective studies to quantify myocardial injury in unselected patients with COVID-19 who underwent systematic cardiac biomarker testing. Moreover, previous data show that increased troponin is common in hospitalized patients with acute illnesses irrespective of the presentation and is a predictor of poor outcomes (14). As with previous reports, no control group was included to explore if myocardial injury in COVID-19 was as common in patients with other acute illnesses such as pneumonia (15). Whether the prognosis of patients with myocardial injury in COVID-19 is worse or comparable to other acute illnesses is not yet known. Finally, it is unclear whether myocardial injury in COVID-19 has any lasting functional consequences for those who survive the initial illness.

Faced with major uncertainty during a rapidly escalating global pandemic, clinicians and researchers have sought to understand the consequences of SARS-CoV-2 on the heart. Biomarkers such as high-sensitivity cardiac troponin have provided invaluable insights into the risk of deterioration and in-hospital death. However, it is increasingly clear due to the work of De Michieli and others that, in most patients, the cardiac consequences of COVID-19 are indirect, with myocardial injury due to preexisting disease or acute severe illness. The lessons learned in understanding this new threat should inform the wider use of cardiac biomarkers in acute care. If we recognize that cardiac troponin increases are often not due to myocardial infarction and carefully consider the likely mechanism of myocardial injury, we may be able to improve care for patients when they are at their most vulnerable.

## Employment or Leadership

None declared.

## Consultant or Advisory Role

None declared.

## Stock Ownership

None declared.

## Honoraria

N.L. Mills has received honoraria from Abbott Diagnostics, Roche Diagnostics, Siemens Healthineers, and LumiraDx.

## Expert Testimony

None declared.

## Patents

None declared.

**Other Remuneration:** N.L. Mills, personal fees from Abbott Diagnostics, Siemens Healthineers, Roche Diagnostics, and LumiraDx.
